# Modulation of Auditory Evoked Magnetic Fields Elicited by Successive Frequency-Modulated (FM) Sweeps

**DOI:** 10.3389/fnhum.2017.00036

**Published:** 2017-02-06

**Authors:** Hidehiko Okamoto, Ryusuke Kakigi

**Affiliations:** ^1^Department of Integrative Physiology, National Institute for Physiological SciencesOkazaki, Japan; ^2^Department of Physiological Sciences, Graduate University for Advanced StudiesHayama, Japan

**Keywords:** adaptation, auditory cortex, brain, frequency modulation (FM), habituation, human, magnetoencephalography (MEG)

## Abstract

In our daily life, we are successively exposed to frequency-modulated (FM) sounds that play an important role in speech and species-specific communication. Previous studies demonstrated that repetitive exposure to identical pure tones resulted in decreased neural activity. However, the effects of repetitively presented FM sounds on neural activity in the human auditory cortex remain unclear. In the present study, we used magnetoencephalography to investigate auditory evoked N1m responses elicited by four successive temporally repeated and superimposed FM sweeps in three sequences: (1) four FM sweeps were identical, (2) four FM sweeps had the same FM direction and rate, but different carrier frequencies, (3) four FM sweeps differed with respect to the FM rate and/or direction and their carrier frequencies. In contrast to our expectations, the results obtained demonstrated that N1m responses were maximal when the four FM sweeps were identical and minimal when they were distinct. These results suggest that the neural processing of repetitive FM sweeps in the human auditory cortex may differ from that of repetitive pure tones.

## Introduction

In daily life, we are continually exposed to repetitive sound signals, such as the ticking of a clock, that are irrelevant for listeners. However, these sound signals are easily ignored and neural resources are conserved for unexpected changes in the acoustic environment (Bregman, 1990). The decrements elicited in neural activity by repetitive auditory signals appear to play an important role in this process. Previous studies on humans demonstrated that the first pure tone elicits a maximal N1/ N1m response, which is a major deflection in electroencephalography, or magnetoencephalography (MEG) waveforms with a latency of approximately 0.1 s, and subsequent tones elicit smaller N1/N1m responses (see a review, Näätänen and Picton, 1987). The decrement in neural activity elicited by repetitive stimuli is not limited to the auditory modality (Butler, 1968; Fruhstorfer et al., 1970; Budd et al., 1998; Uhlig et al., 2016), it is also observed in visual (Rugg et al., 1995; Buckner et al., 1998; Kourtzi and Kanwisher, 2000) and somatosensory modalities (Allison, 1962; Angel et al., 1985; Otsuru et al., 2011) in the human brain.

Even though adaptive phenomena may be observed over long timescales such as the evolution of a species, we focused on the neural adaptation that occurs within the timescales of hundreds of ms to seconds in the present study. Using auditory oddball paradigms, in which a high-probability sound (“standard”) and low-probability sound (“deviant”) were randomly presented, Ulanovsky et al. (2003) demonstrated that neurons in the cat primary auditory cortex showed stronger neural activity corresponding to “deviant” tones rather than “standard” tones because of the neural mechanism underlying stimulus-specific adaptation. Excitatory and inhibitory neural networks within the central auditory pathway appear to enhance the neural processing of stimulus-specific adaptation (Taaseh et al., 2011; Malmierca et al., 2015) and may contribute to better auditory novelty detection (Malmierca et al., 2014; Chen et al., 2015). However, most of the previous studies that investigated stimulus-specific adaptation used simple pure tones as sound stimuli; therefore, neural decrements induced by repetitive complex sound signals remain elusive.

The neural encoding of frequency modulated (FM) sweeps appears to differ, at least partially, from that of pure tones. Pure tones vibrate specific portions of the basilar membrane according to the tonotopic map in the cochlea and the vibration patterns of the basilar membrane remain constant during the pure tone presentation (Reale and Imig, 1980; Schreiner and Langner, 1988; Robles and Ruggero, 2001). In contrast, FM sweeps change their vibration patterns of the basilar membrane over time. Therefore, in order to process FM sound signals, their carrier frequencies as well as FM rates and directions need to be analyzed simultaneously (Deboer and Dreschler, 1987; Eggermont, 1998; Zatorre et al., 2002; Obleser et al., 2008). Previous studies showed that FM sweeps caused stronger neural activity than pure tones in the primary auditory cortex of marmosets (Liang et al., 2002) as well as stronger non-primary auditory area activity than that in the primary auditory cortex in cats (Tian and Rauschecker, 1994, 1998; Heil and Irvine, 1998). Moreover, a recent MEG study measuring the neural activity elicited by temporally repeated and superimposed FM sweeps revealed that lower-rate FM sweeps (1 and 4 octaves *per sec*) elicited larger N1m source strengths and shorter N1m latencies than higher-rate FM sweeps (16 and 64 octaves *per sec*; Okamoto and Kakigi, 2015). FM sweeps in human speech play an essential role in verbal communications. For example, changing the FM direction of the third formant within a human voiced “ba” sound turned speech perception into “ga” (Liberman et al., 1957). In order to follow speech, it is inevitable for humans to execute the proper neural processing of FM sweeps that repeatedly appear in daily conversations; however, the neural responses elicited by repeatedly presented FM sweeps remain elusive in the human auditory cortex.

**Figure 1:**
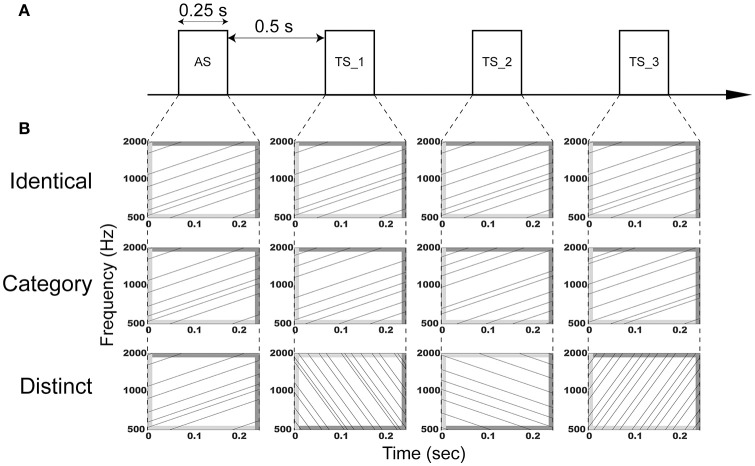
**Schematic display of the auditory stimulation. (A)** Adaptor stimuli (AS) and subsequent test stimuli (TS_1, TS_2, and TS_3) with a duration of 0.25 s were presented with an inter-stimulus interval of 0.5 s. **(B)** AS and TS consisted of six temporally repeating frequency-modulated (FM) tones that traversed an upward or downward direction with a modulation rate of 4 or 16 octaves *per sec*. The dark and light gray areas represent the linear rise- and fall-ramps of the sound signals, respectively. In the “Identical” sequence (upper row), AS, TS_1, TS_2, and TS_3 were identical. In the “Category” sequence (middle row), AS, TS_1, TS_2, and TS_3 were characterized by the same modulation rate and direction, but different carrier frequencies. In the “Distinct” sequence (lower row), AS, TS_1, TS_2, and TS_3 had a distinct modulation rate and/or direction. Exemplary sound files of “Identical,” “Category,” and “Distinct” sequences are available online as Audio 1, Audio 2, and Audio 3 in Supplementary Material, respectively.

The aim of the present study was to investigate the adaptation of auditory evoked N1m responses elicited by four successive FM sweeps using MEG. We used temporally repeated and superimposed FM sweeps that were matched in the spectral domain, but differed in their direction and modulation rate as adaptor stimuli (AS) and test stimuli (TS) (Figure 1) and presented them using an adaptation paradigm, in which trains of four successive FM sweeps were presented in three manners: (1) AS and three consequent TS were completely identical (“Identical” sequence), (2) AS and TS had the same FM direction and rate, but different carrier frequencies (“Category” sequence), (3) AS and TS differed with respect to the FM rate and/or direction and their carrier frequencies (“Distinct” sequence). Crucially, we used overall (i.e., in sum) identical AS and TS between sequences, and thereby controlled neural activity differences caused by different FM sweeps. Similar to the N1m responses elicited by successive pure tones, we hypothesized that the auditory evoked N1m responses eliciting the successive FM sweeps may be the smallest in the “Identical” sequence and the largest in the “Distinct” sequence.

## Materials and methods

### Participants

Thirteen healthy people (nine females; mean ± standard deviation: 25.7 ± 7.4 years) participated in the present study. All participants were right handed [assessed via Edinburgh Handedness Inventory (Oldfield, 1971)] and had no history of otological or neurological disorders. They were fully informed about the study and gave written informed consent for their participation. The study was approved by the Ethics Commission of the National Institute for Physiological Sciences and conformed to The Code of the World Medical Association (Declaration of Helsinki).

### Stimuli and experimental design

The experimental design is schematically displayed in Figure 1. Adaptor stimuli (AS) were followed by three test stimuli (TS). AS and TS had a duration of 0.25 s with 0.01-s linear onset and offset ramps (sampling rate: 48,000 Hz). They were temporally repeated and superimposed FM sweeps similar to those used in our previous study (Okamoto and Kakigi, 2015). They consisted of six FM tones that traversed an upward or downward direction within the 500–2000 Hz frequency range with a modulation rate of 4 or 16 octaves *per sec*, resulting in four FM sweeps (FM_up_04, FM_up_16, FM_down_04, and FM_down_16). FM_up_04 and FM_up_16 had 25- and 6.25-ms linear rise ramps starting at 500 Hz and 25- and 6.25-ms linear fall ramps ending at 2000 Hz, whereas FM_down_04 and FM_down_16 had 25- and 6.25-ms linear rise ramps starting at 2000 Hz and 25- and 6.25-ms linear fall ramps ending at 500 Hz, respectively (Figure 1). The rise and fall ramps of the FM tones simultaneously started in order to minimize the sound envelope change.

We prepared 48 FM sweeps that were characterized by different initial spectral components in each FM type (FM_up_04, FM_up_16, FM_down_04, or FM_down_16), resulting in 192 AS and TS. We presented AS and three successive TS (TS_1, TS_2, and TS_3) with an inter-stimulus interval of 0.5 s in specific manners (Figure 1). In the “Identical” sequence, four identical sounds were successively presented (Audio 1 in Supplementary Material). In the “Category” sequence, AS and TS belonging to the same FM type (FM_up_04, FM_up_16, FM_down_04, or FM_down_16), but differing with respect to carrier frequencies were presented (Audio 2 in Supplementary Material). In the “Distinct” sequence, four distinct FM types with different carrier frequencies were successively presented (Audio 3 in Supplementary Material). Each FM sweep appeared only once at a given position (AS, TS_1, TS_2, or TS_3) in each sequence (“Identical,” “Category,” or “Distinct”). Therefore, total sound inputs were counter-balanced between positions and between sequences. The “Identical,” “Category,” and “Distinct” sequences were pseudo-randomly presented. The silent interval between the preceding TS_3 and the subsequent AS was 2.5 s.

All FM sweeps were adjusted to have equal energy and presented by insert earphones (E-ARTONE 3A, Aearo Company Auditory Systems, Indianapolis, IN) through 1.5-m plastic tubes attached to foam plugs (E-A-RLINK, Aearo Company Auditory Systems, Indianapolis, IN). Prior to starting the MEG experiment, we examined each participant's hearing threshold for FM_up_04 in each ear. During the MEG recording session, TS were diotically presented at an intensity of 50 dB more than the individual hearing threshold. Participants were comfortably seated upright and were instructed to watch a self-chosen silent movie with captions in order to keep them alert during the MEG measurement.

### Data acquisition and analysis

Auditory evoked magnetic fields were recorded with a helmet-shaped 204-channel whole head planar-type gradiometer (Vector-view, ELEKTA, Neuromag, Helsinki, Finland) located in a magnetically shielded and acoustically quiet room. Prior to the MEG recording, four head position indicator coils were attached to the participant's scalp. A 3D digitizer (Polhemus Inc., Colchester, VT) was used to measure the locations of head position indicator coils and three anatomical landmarks, nasion, and bilateral pre-auricular points, and head shapes. A current was fed to the four head position indicator coils and the resulting magnetic fields were used to assess the head position of the participant with respect to the MEG dewar. Signals were filtered online using a bandpass of 0.1–200-Hz and digitized at 1000 Hz. The magnetic fields starting 0.15 s prior to the sound onset and ending 0.15 s after the sound offset were averaged selectively for each position (AS, TS_1, TS_2, and TS_3) in each sequence (“Identical,” “Category,” or “Distinct”) irrespective of the FM types (FM_up_04, FM_up_16, FM_down_04, or FM_down_16). Epochs containing amplitude values of >3 pT/cm were discarded as artifact-contaminated epochs.

In order to analyze auditory evoked N1m responses, we estimated N1m source locations and orientations by means of two single equivalent current dipoles (one for each hemisphere) using the brain electric source analysis software package (BESA Research 5.3.7, BESA GmbH, Germany). In the N1m source analysis, the grand-averaged magnetic field signals elicited by all FM sweeps after artifact rejection were 30 Hz low-pass filtered (zero-phase shift Butterworth filter, 24 dB/oct), and the baseline was corrected relative to the 0.1-s pre-stimulus interval. The peak N1m response was initially identified as the maximal root-mean square value of the global field power of all the sensors within the time interval from 0.075 to 0.15 s after the test stimulus onset. The single equivalent current dipole model was used for source locations and orientations based on the 0.01-s time window around the N1m peak using all the sensors for each participant and hemisphere. The locations and orientations of the equivalent current dipoles corresponding to the N1m responses were individually determined in a Cartesian coordinate system with the medial—lateral axis connecting the pre-auricular points, the posterior—anterior axis passing through the nasion perpendicular to the medial—lateral axis, and the inferior—superior axis orthogonal to the medial—lateral and posterior-lateral axes. The estimated N1m source location and orientation were used to calculate the source strength waveform as a spatial filter in each participant and hemisphere (Tesche et al., 1995). Thereafter, we obtained the maximal N1m source strengths and N1m latencies in each sequence and each position based on the calculated source strength waveforms.

In order to examine hemispheric differences in the N1m source strengths elicited by FM sweeps, we calculated the mean N1m source strengths elicited by AS and TS in each hemisphere and each participant. We then performed planned comparisons (paired two-tailed *t*-tests) between the left and right hemispheres. Thereafter, in order to avoid the source strength difference between participants and hemispheres, the source strengths of the N1m responses elicited by TS_1, TS_2, and TS_3 were individually normalized with respect to the N1m source strength elicited by AS in each sequence and each hemisphere. Normalized N1m source strengths and N1m latencies were evaluated by means of three-way repeated-measures analysis of variances (ANOVAs) using Hemisphere (Left vs. Right), Sequence (“Identical,” “Category,” and “Distinct”), and Position (TS_1, TS_2, and TS_3) as factors. Thereafter, Bonferroni-corrected paired *t*-tests were performed for *post hoc* multi-comparisons. Statistical analyses were performed using SPSS (V 21, IBM Corp.). We applied the Shapiro-Wilk test to establish whether data were normally distributed.

## Results

**Figure 2:**
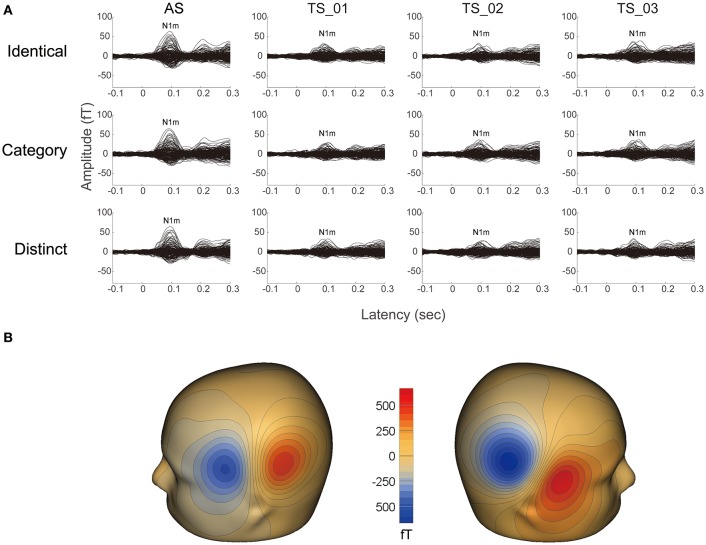
**Examples of individual magnetic waveforms. (A)** Each column represents the auditory evoked fields elicited by the adaptor stimuli (AS) and three test stimuli (TS_1, TS_2, and TS_3) (from left to right) in the “Identical” (upper row), “Category” (middle row), and “Distinct” (lower row) sequences, respectively. **(B)** Iso-contour maps of the magnetic fields at the N1m latency of the grand-averaged magnetic waveforms across AS, TS_1, TS_2, and TS_3. The magnetic contour maps show clear dipolar patterns above the left (left panel) and right (right panel) hemispheres. Red and blue contour lines represent the outbound and inbound flows of magnetic fields from and into the brain.

It was possible to average a sufficient number of trials for each condition in 13 participants after artifact rejection (mean ± standard deviation: 189.8 ± 3.0), and clear auditory evoked N1m responses were obtained under each condition (cf. Figure 2). The goodness-of-fit of the underlying dipolar source models for the averaged MEG waveforms of all the gradiometers was more than 90% in all participants (mean ± standard deviation: 96.4 ± 1.8%).

**Figure 3:**
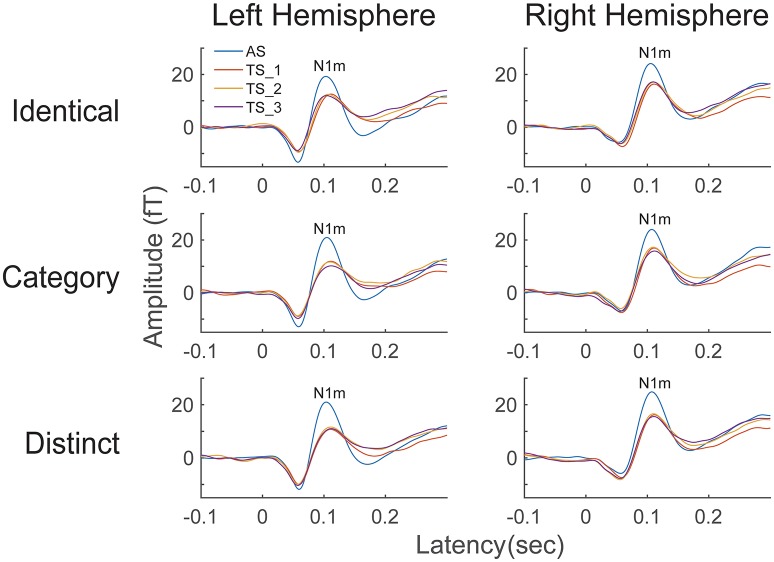
**Source strength waveforms calculated at the N1m generator averaged across 13 participants in the left and right hemispheres**. Top, middle, and bottom rows represent the source strength waveforms in the “Identical,” “Category,” and “Distinct” sequences, respectively. Each colored line indicates the position of the test stimuli (TS): The 1st (AS), 2nd (TS_1), 3rd (TS_2), and 4th (TS_3) FM sweeps were colored as blue, red, yellow, and purple, respectively (see legends in the left upper corner).

The time courses of the N1m source strengths (from −0.1 to +0.3 s) grand-averaged across all participants (*N* = 13) are displayed in Figure 3. An N1m response with a latency of approximately 0.1 s is clearly shown. According to the Shapiro-Wilk test, data were normally distributed using the mean N1m source strengths elicited by AS and TS in each hemisphere as a dependent variable (Left Hemisphere: *p* = 0.515; Right Hemisphere: *p* = 0.599). The planned comparison applied to the N1m source strength revealed a significant difference between hemispheres [*t*_(1, 12)_ = 2.53, *p* = 0.027]. N1m responses had larger source strengths in the right than in the left hemisphere.

**Figure 4:**
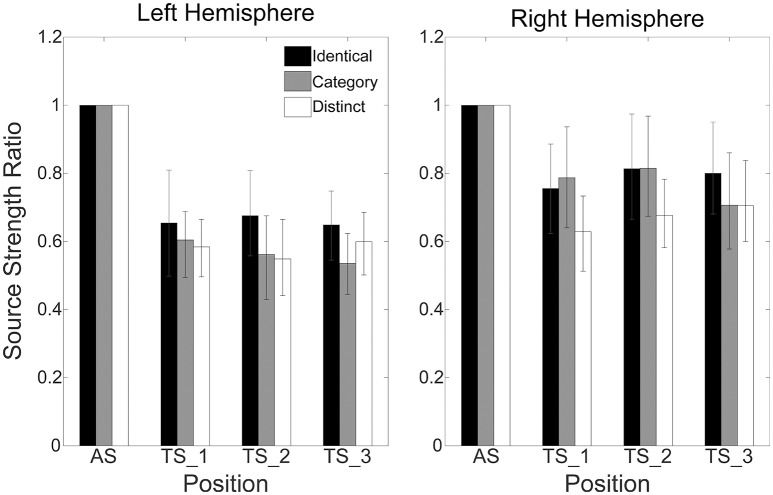
**Group means (*N* = 13) of normalized N1m source strengths in the left (left panel) and right (right panel) hemispheres elicited by the 1st (AS), 2nd (TS_1), 3rd (TS_2), and 4th (TS_3) trains of four successive FM sweeps, including error bars denoting 95% confidence intervals**. Black, gray, and white bars denote the “Identical,” “Category,” and “Distinct” sequences, respectively.

**Figure 5:**
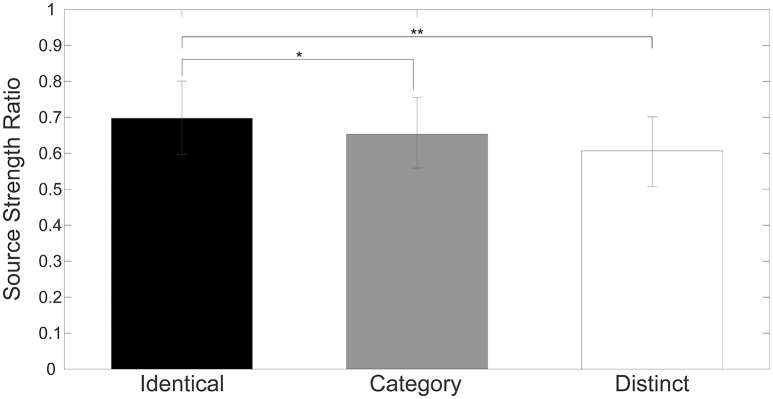
**Group means (*N* = 13) of mean normalized N1m source strengths elicited by TS_1, TS_2, and TS_3 in both hemispheres with error bars denoting 95% confidence intervals**. Black, gray, and white bars denote the “Identical,” “Category,” and “Distinct” sequences, respectively. [^*^ *p* < 0.05, ^**^ *p* < 0.01 (Bonferroni-corrected)].

Figure 4 shows mean normalized N1m source strengths in each sequence (“Identical,” “Category,” or “Distinct”), in each hemisphere (Left or Right), and in each position (AS, TS_1, TS_2, or TS_3) together with the corresponding 95% confidence intervals obtained by boot-strap resampling tests (iteration = 100,000). According to the Shapiro-Wilk test, data were normally distributed using the mean normalized N1m source strengths in each sequence, hemisphere, and position as a dependent variable (“Identical_Left_TS_1”: *p* = 0.87; “Identical_Left_TS_2”: *p* = 0.91; “Identical_Left_TS_3”: *p* = 0.26; “Identical_Right_TS_1”: *p* = 0.97; “Identical_Right_TS_2”: *p* = 0.22; “Identical_Right_TS_3”: *p* = 0.16; “Category_Left_TS_1”: *p* = 0.18; “Category_Left_TS_2”: *p* = 0.83; “Category_Left_TS_3”: *p* = 0.69; “Category_Right_TS_1”: *p* = 0.17; “Category_Right_TS_2”: *p* = 0.23; “Category_Right_TS_3”: *p* = 0.27; “Distinct_Left_TS_1”: *p* = 0.42; “Distinct_Left_TS_2”: *p* = 0.67; “Distinct_Left_TS_3”: *p* = 0.40; “Distinct_Right_TS_1”: *p* = 0.63; “Distinct_Right_TS_2”: *p* = 0.91; “Distinct_Right_TS_3”: *p* = 0.38). The three-way repeated-measures ANOVA applied to normalized N1m source strengths revealed significant main effects for Sequence [*F*_(2, 24)_ = 11.26, *p* < 0.001] and Hemisphere [*F*_(1, 13)_ = 4.74, *p* = 0.05], and a significant interaction between Sequence and Hemisphere [*F*_(2, 24)_ = 6.87, *p* = 0.004]; however, no significant main effect for Position [*F*_(2, 24)_ = 1.38, *p* = 0.27] was found. The Bonferroni-corrected *post hoc* multi-comparisons revealed significant differences between “Identical” and “Category” [*t*_(12_) = 3.09, *p* = 0.028] and between “Identical” and “Distinct” [*t*_(12)_ = 4.22, *p* = 0.004]; however, no significant difference between “Category” and “Distinct” [*t*_(12)_ = 2.27, *p* = 0.127] was found, as shown in Figure 5.

**Figure 6:**
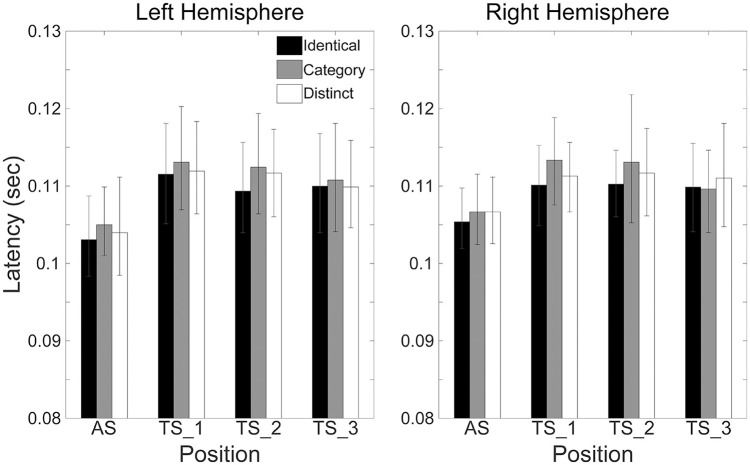
**Group means (*N* = 13) of N1m latencies in the left (left panel) and right (right panel) hemispheres elicited by the 1st (AS), 2nd (TS_1), 3rd (TS_2), and 4th (TS_3) trains of four successive FM sweeps, including error bars denoting 95% confidence intervals**. Black, gray, and white bars denote the “Identical,” “Category,” and “Distinct” sequences, respectively.

Figure 6 shows the mean N1m latencies in each hemisphere (Left or Right), in each sequence (“Identical,” “Category,” or “Distinct”), and in each position (AS, TS_1, TS_2, or TS_3) together with the corresponding 95% confidence intervals obtained by boot-strap resampling tests (iteration = 100,000). According to the Shapiro-Wilk test, data were normally distributed using the N1m latencies in each sequence, hemisphere, and position as a dependent variable (“Identical_Left_TS_1”: *p* = 0.991; “Identical_Left_TS_2”: *p* = 0.11; “Identical_Left_TS_3”: *p* = 0.51; “Identical_Right_TS_1”: *p* = 0.97; “Identical_Right_TS_2”: *p* = 0.43; “Identical_Right_TS_3”: *p* = 0.35; “Category_Left_TS_1”: *p* = 0.57; “Category_Left_TS_2”: *p* = 0.42; “Category_Left_TS_3”: *p* = 0.91; “Category_Right_TS_1”: *p* = 0.86; “Category_Right_TS_2”: *p* = 0.28; “Category_Right_TS_3”: *p* = 0.95; “Distinct_Left_TS_1”: *p* = 0.62; “Distinct_Left_TS_2”: *p* = 0.33; “Distinct_Left_TS_3”: *p* = 0.78; “Distinct_Right_TS_1”: *p* = 0.54; “Distinct_Right_TS_2”: *p* = 0.28; “Distinct_Right_TS_3”: *p* = 0.68). The three-way repeated-measures ANOVA applied to N1m latencies revealed neither a significant main effect [Sequence: *F*_(2.24)_ = 1.97, *p* = 0.16; Hemisphere: *F*_(1, 12)_ = 0.01, *p* = 0.93; Position: *F*_(2, 24)_ = 2.05, *p* = 0.15] nor a significant interaction between them. N1m latencies did not significantly differ between hemispheres or sequences.

## Discussion

In the present study, we investigated auditory evoked N1m responses elicited by four successive, temporally repeated, and superimposed FM sweeps by means of MEG. The results obtained demonstrated that the N1m source strengths elicited by TS were significantly influenced by the sequence of the FM sweep presentation. In contrast to our hypothesis, the successive presentation of four identical FM sweeps (“Identical” sequence) resulted in maximal normalized N1m source strengths, whereas those in the “Distinct” sequence in which four FM sweeps differed both in the FM modulation rate and/or direction and in carrier frequencies elicited minimal normalized N1m source strengths (Figures 4, 5). Moreover, the normalized N1m source strengths elicited in the “Category” sequence in which the FM sweeps had the same FM modulation rate and direction, but different carrier frequencies were around the middle of those elicited in the “Identical” and “Distinct” sequences.

The amplitude and latency of the N1m response is known to be influenced by the spectral components of the test sound signals (Pantev and Lutkenhöner, 2000; Roberts et al., 2000); however, in the present study, we used temporally repeated and superimposed FM sweeps that were balanced with respect to the spectral components (Figure 1). Moreover, we prepared 48 FM sweeps for each sound type (FM_up_04, FM_up_16, FM_down_04, or FM_down_16), resulting in 192 FM sweeps. We presented them once in each position (AS, TS_1, TS_2, or TS_3) in each sequence (“Identical,” “Category,” or “Distinct”). Therefore, the total bottom-up sound inputs were identical between sequences; only the sound presentation patterns differed between sequences (“Identical,” “Category,” or “Distinct”), and thus, may have had a significant impact on N1m responses. The results obtained suggest that neural adaptation in the human auditory cortex is sensitive to the rate and direction of FM sweeps and their carrier frequencies.

The results obtained appear to be contradictory to previous findings showing that the N1/ N1m responses elicited by repetitive identical pure tones were smaller than those elicited by successive distinct pure tones (Butler, 1968; Sams et al., 1985; Lagemann et al., 2012). Previous studies showed that neurons in the auditory cortex are sensitive to the rate and/or direction of FM sweeps in animals (Mendelson and Cynader, 1985; Heil and Scheich, 1992; Heil et al., 1992; Mendelson et al., 1993; Nelken and Versnel, 2000; Tian and Rauschecker, 2004; Godey et al., 2005; Atencio et al., 2007; Brown and Harrison, 2009; Trujillo et al., 2011) and humans (Hall et al., 2000, 2002; Hsieh et al., 2012; Joanisse and Desouza, 2014; Okamoto and Kakigi, 2015). The neural processing of repetitive FM sweeps in the human auditory cortex appears to differ from that of repetitive pure tones. Previous MEG studies using a two-tone adaptation paradigm supported our results by demonstrating that auditory evoked fields elicited by subsequent FM sweeps were larger when the preceding and subsequent FM sweeps were identical than when they had opposite FM directions; however, this effect was not observed in repetitive complex tones (Heinemann et al., 2010, 2011). These findings were consistent with our results demonstrating maximal normalized N1m source strengths elicited by four repetitive identical FM sweeps in the “Identical” sequence. Moreover, we found that normalized N1m source strengths in the “Category” sequence were significantly smaller than those in the “Identical” sequence. In the “Category” sequence, even though they had the same FM rate and direction, the four successive TS were characterized by different carrier frequencies, leading to different sensations in pitch and timber. Exposure to four successive sounds with the same pitch and timber may have resulted in larger N1m responses in the “Identical” sequence than in the “Category” sequence.

Inhibitory and excitatory neural circuits appear to contribute to neural adaptation in the auditory cortex (Whitmire and Stanley, 2016). Recent advances in genetic technology make it possible to activate and inactivate cell-type-specific neural circuits in behaving animals (Luo et al., 2008). Recent studies (Aizenberg et al., 2015; Natan et al., 2015) demonstrated that parvalbumin-positive interneurons inhibited neural responses to “standard” tones and “deviant” tones, whereas somatostatin-positive interneurons specifically reduced excitatory neural activity to “standard” tones. Moreover, parvalbumin-positive neurons and somatostatin-positive neurons appear to play major roles in fast responding inhibition and slow and long-lasting inhibition, respectively (Li et al., 2014, 2015). This slower late frequency-specific inhibitory neural activity (0.2–0.4 s) may account for the adaptation of N1m responses elicited by repetitive pure tones. However, we herein used temporally repeated and superimposed FM sweeps as AS and TS, which were matched in the spectral domain, and changed their frequency components over time. Therefore, unlike repetitive pure tones, somatostatin-positive interneurons may not effectively inhibit the neural activity elicited by repetitive FM sweeps. Interactions between multiple excitatory-inhibitory neural circuits including parvalbumin-positive interneurons and somatostatin-positive interneurons may lead to the different sequence effects on N1m responses elicited by repetitive pure tones and FM sweeps.

In the present study, the auditory evoked N1m responses elicited by TS in all sequences (“Identical,” “Category,” and “Distinct”) were smaller than those elicited by AS (Figures 2–4). This result appears to be contradictory to previous findings showing that the 2nd FM sweep elicited larger N1m responses than the 1st FM sweep (Heinemann et al., 2010). The main reason for this inconsistency appears to be the difference in inter-stimulus intervals between FM sweeps. We adopted a longer inter-stimulus interval (0.5 s) between sounds than that used in the previous study (0.2 s). Previous electroencephalography and MEG studies reported that inter-stimulus intervals shorter than 0.5 s may cause enhanced N1/ N1m responses (Budd and Michie, 1994; Loveless et al., 1996). Moreover, in the present study, we corrected the baseline relative to a 0.1-s pre-stimulus interval for each position (1st, 2nd, 3rd, and 4th), whereas Heinemann et al. (2010) applied the baseline correction only once relative to a 0.1-s pre-stimulus period before the 1st FM sweep. These differences in the experimental design appear to have led to differences in the results obtained.

The results of the present study showed that N1m source strengths elicited by AS and TS and normalized Nm source strengths elicited by TS were significantly larger in the right than in the left hemisphere. Moreover, a significant interaction between Sequence and Hemisphere in the present study indicated that neural modulation induced by successive FM sweeps might differ between hemispheres. The functional hemispheric asymmetries of the human auditory cortex are often observed in higher stage auditory processing. Previous neuroimaging studies revealed that the right hemisphere played an important role in listening to music (Zatorre et al., 1994; Griffiths et al., 1999), whereas the left hemisphere played a major role in speech processing (Eulitz et al., 1995; Alho et al., 1998; Belin et al., 2000; Szymanski et al., 2001). These functional hemispheric asymmetries may not be limited to complex natural sounds, but may originate from the neural processing of basic acoustic features. Positron emission tomography (Zatorre and Belin, 2001) and functional MRI (Jamison et al., 2006) studies demonstrated that the right hemisphere plays a dominant role in spectral processing. Previous MEG studies (Heinemann et al., 2011; Okamoto and Kakigi, 2015) also revealed that the N1m responses elicited by FM sweeps were larger in the right than in the left hemisphere. The right hemispheric dominance for FM sweep processing observed in the present study is also consistent with previous findings demonstrating that auditory cortex lesions in the right hemisphere caused severe impairments in detecting the frequency modulation of test sounds, whereas lesions in the left did not cause such an impairment in animals (Wetzel et al., 1998; Rybalko et al., 2006) or humans (Johnsrude et al., 2000).

In conclusion, using appropriately designed FM sweeps that were balanced between sequences and positions with respect to the total bottom-up sound inputs, we herein clearly demonstrated that the rate and/or direction of FM sweeps and their carrier frequencies influenced the N1m responses elicited by the successive FM sweeps. The results obtained suggest that the modulation of neural activity caused by successive FM sweeps differs from that of successive pure tones and may contribute to the efficient encoding of daily speech signals, which typically contain rapid repetitions of FM sweeps.

## Author contributions

HO conceived and designed the study; HO performed experiments; HO analyzed data; HO interpreted results of experiments; HO prepared figures; HO drafted manuscript; HO and RK edited and revised manuscript; HO and RK approved final version of manuscript.

## Funding

This work has been supported by the “Japan Society for the Promotion of Science for Young Scientists (26861426).”

### Conflict of interest statement

The authors declare that the research was conducted in the absence of any commercial or financial relationships that could be construed as a potential conflict of interest.
